# Portable and wearable dialysis devices for the treatment of patients with end-stage kidney failure: Wishful thinking or just over the horizon?

**DOI:** 10.1007/s00467-014-2968-3

**Published:** 2014-10-21

**Authors:** Andrew Davenport

**Affiliations:** UCL Centre for Nephrology, Royal Free Hospital, University College London Medical School, Rowland Hill Street, London, NW3 2PF UK

**Keywords:** Wearable, Portable, Haemodialysis, Haemofiltration, Peritoneal dialysis

## Abstract

Dialysis is a life-sustaining treatment for patients with end-stage kidney disease. In a different context, for many patients this treatment is the focal point around which their life revolves, not only due to the time spent travelling to and from treatment sessions and the time dedicated to the dialysis treatment itself, but also due to the accompanying dietary and fluid restrictions and medication burden. Wearable and portable dialysis devices could potentially improve patient quality of life by allowing patients to continue with their daily activities of life while undergoing dialysis, as well as by loosening—or removing entirely—dietary and fluid restrictions and reducing pill burden. Advances in nanotechnology manufacturing coupled with advances in electronics and miniaturisation have allowed a new generation of wearable and portable dialysis devices to be developed which are now undergoing large animal and patient clinical trials. We are therefore potentially at a new dawn in the treatment of dialysis patients with the first generation of wearable and portable dialysis devices, which may well revolutionise the treatment and quality of life for patients with end-stage kidney disease.

## Introduction

Haemodialysis (HD) was first used by Wilhem Kolff to treat patients with acute kidney injury during the Second World War [1]. However it was only in the 1960s following advances in vascular access, dialysers [2] and dialysis machine design [3] that HD started to become available as a treatment for patients with advanced chronic kidney disease (CKD). The many limitations on patient life style at that time, particularly the strict dietary and fluid restrictions required, were readily appreciated by the early pioneers of HD therapies, who started a search to develop portable and wearable HD devices [4–6]. However the technology available at that time restricted development and, consequently, enthusiasm for developing a wearable device waned.

## Why develop a wearable or implantable dialysis device ?

Home HD offers many advantages over centre-based dialysis, and the newer dialysis machine designs reduce the time spent in preparing and cleaning the dialysis machine. However unless patients dialyse overnight, they still potentially lose productive time while attached to the dialysis machine. Thus, a wearable or implantable device could potentially provide patients with the freedom to work and perform their activities of daily living while dialysing, and allow greater dietary choices [7, 8].

It could be argued that a portable and wearable dialysis device—in the form of peritoneal dialysis (PD)—has already been developed. However, patients either have to perform three to four exchanges per day with continuous ambulatory PD or connect themselves to an automated overnight cycler which although transportable requires a mains electrical supply and fresh dialysate. A PD system that recycles dialysate would potentially be more eco-friendly, and fewer connections and disconnections could potentially reduce the risk of peritonitis, the commonest cause of PD technique failure [9, 10].

**Fig. 1:**
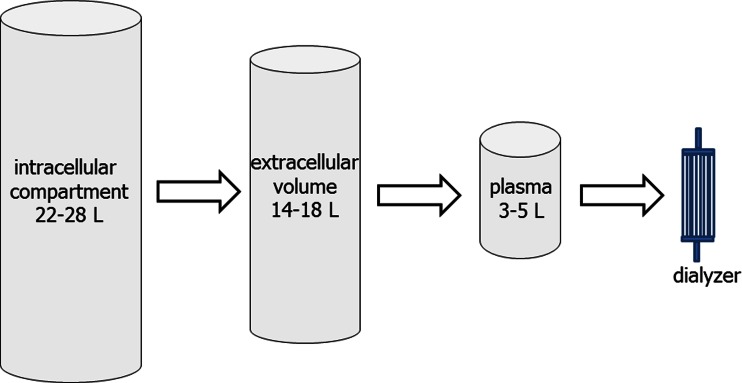
Patients with chronic kidney disease fail to adequately excrete the products of cellular metabolism. The volume of the intracellular compartment exceeds that of the plasma volume, and the clearance of most middle-sized and charged solutes by the dialyser is time dependent, so favouring more effective clearance with a continuous dialysis system

HD is an efficient treatment for removing small water-soluble solutes, but trials have consistently shown that simply increasing urea clearance does not lead to improved patient survival for patients with both acute and chronic kidney failure [11–13]. While adding convective losses to the diffusive clearance of conventional HD can increase the clearance of phosphate and other middle molecular-weight solutes [14, 15], as these are predominantly intracellular, their extracorporeal clearance is more dependent on time than modality (Fig. 1) [16].

More recently, high volume post-dilutional on-line haemodiafiltration treatments have been compared to standard HD[17–20]. The results appear to indicate benefit in terms of patient outcomes [17]: taken individually these trials have a number of potential confounders [18–20], but taken together, higher volume haemodiafiltration does appear to offer improved patient survival [17]. Other trials on HD, involving either frequent HD sessions daily but each of shorter duration or longer nocturnal HD sessions, have not shown the expected improvements from a greater amount of dialysis treatment [21, 22]. Indeed, a increase number of HD sessions led to a quicker loss of residual renal function, so perhaps negating the benefits of additional dialysis [21, 22]. However, the combination of more frequent dialysis sessions and haemodiafiltration [5× week predilution convective flow of 18–27 l/m^2^ body surface area (BSA)] in children demonstrated not only excellent biochemical results but also highly significant catch-up growth that was much better than that observed following transplantation [23]. Although this treatment option is an advance, the question arises as to whether dialysis centres have the logistics to provide more frequent haemodialfiltration treatments and equally whether children and their parents are prepared to commit so much time to the treatment. A wearable dialysis device could potentially be a solution to this paradox by combining longer treatment times while allowing patients greater freedom [24].

## The new generation of wearable dialysis devices

### PD devices

The current generation of wearable dialysis devices had to overcome to two important basic design problems: firstly, to operate using powerful small light-weight battery-powered pumps and, secondly, to avoid reliance on fresh dialysate by developing sorbent technology to re-use spent dialysate [25]. The developers of the Vicenza wearable artificial kidney (ViWAK) proposed using a standard fresh glucose-based dialysate that was to be instilled each morning, allowed to dwell for 2 h and then continuously recycled through a dual lumen PD catheter. The dialysate effluent would be pumped first through a filter to remove proteins and then through a series of sorbent filters, followed by a degassing chamber before returning to the patient [26]. In the evening the patient would drain out the dialysate and instil a fresh bag of 7.5 % icodextrin to aid solute clearance and volume control. Such a system as described would require the patient to perform two standard PD exchanges per day. Due to this limitation and the costs of replacing the sorbents each day, the ViWAK has not proceeded from laboratory to clinical trials.

**Fig. 2:**
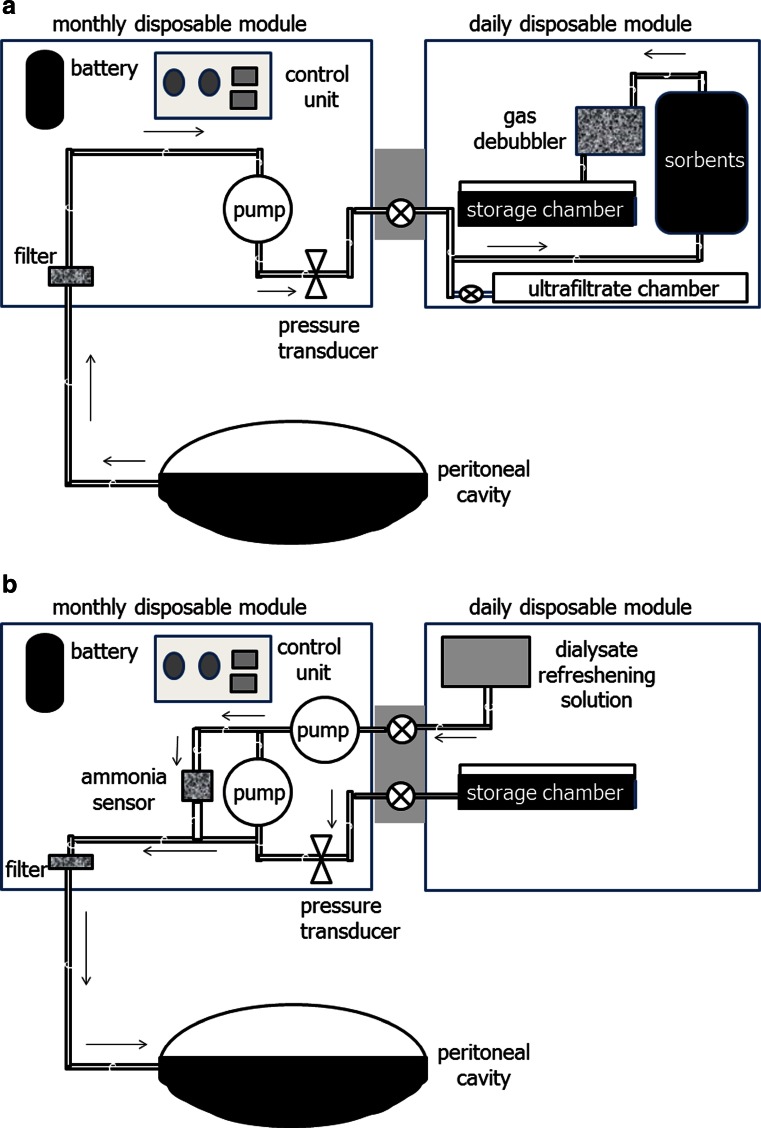
The automated wearable artificial kidney (AWAK) has a discontinuous flow of peritoneal dialysate. **a** Outflow circuit with spent dialysate effluents pumped through a fibrin filter and sorbents and then through a degassing chamber before being retained in a storage chamber. There is a separate collecting chamber for ultrafiltrate. **b** The AWAK in-flow circuit with spent dialysate refreshed by the addition of glucose, bicarbonate and electrolytes before being passed through an ammonia sensor and then pumped back into the patient

David Lee and Marty Roberts worked for many years on developing a wearable continuous peritoneal dialysis device [27]. Their current version, the automated wearable artificial kidney (AWAK) is based on regenerating spent PD effluent [28]. As the AWAK uses a standard single lumen PD catheter, then peritoneal dialysate either flows into or out from the patient (Fig. 2a, b), and as such there has to be a chamber to store peritoneal dialysate. The AWAK device comprises two modules, one designed to be changed on a daily basis and the other to be changed monthly [29].

Sorbents can be subclassified into those which either adsorb or absorb molecules. Adsorption is when molecules adhere to the surface of the adsorbent, whereas absorption is when molecules permeate the sorbent and are subsequently taken up by it. In addition, whereas some sorbents take up molecules until they become saturated, others act primarily by exchanging one molecule for another [30]. For example, many sorbents use activated microporous carbons which both adsorb and absorb many compounds, including heavy metals, oxidants, chloramines, creatinine, other organic species, middle molecules, including beta 2 microglobulin, and also the protein-bound solutes indoxyl-sulphate and* p*-aminohippurate [31]. Urea is cleared enzymatically in the AWAK using urease, which converts urea into ammonium carbonate, producing ammonia and carbon dioxide. As ammonia is toxic, the sorbent system has to contain compounds designed to remove ammonia [32]. Zirconium phosphate will readily adsorb ammonium, but also potassium, calcium, magnesium and other cations and metals. However, when zirconium phosphate adsorbs these molecules they are exchanged, thereby releasing hydrogen and to a lesser extent sodium ions. As hydrogen ion release is not desirable, yet another sorbent is required to remove these hydrogen ions. Zirconium carbonate will absorb hydrogen ions, along with phosphate, fluoride and heavy metals, but once again adsorption is by exchange, so releasing bicarbonate, acetate and to a lesser extent sodium [33].

Although azotaemic toxins are removed by the sorbents, there will also be some changes in electrolytes. Glucose and lactate will have been lost through diffusion from the peritoneal cavity so the dialysate will need to be refreshed by adding glucose, bicarbonate and electrolytes [34] (Fig. 2b). The refreshed dialysate is then pumped back into the patient after passing through an ammonia sensor. This sensor has been designed as a safety mechanism to detect the very small quantities of ammonia which start to enter the dialysate when the sorbents have neared their capacity to adsorb ammonium, thus warning the patient that fresh sorbents are required. Compared to the conventional PD modality, the AWAK design proposes a tidal protocol with a residual volume of 500–1,000 ml with rapid exchanges of around 250-ml aliquots aiming for exchanges of around 4 l/h [35]. Although the pumps are operated by small light-weight rechargeable batteries, they require recharging overnight.

One of the key decisions to be made in designing a portable or wearable device is to determine the amount of sorbent to be used, as too little sorbent will lead to earlier saturation and sorbent exhaustion with increased frequency of sorbent exchanges, whereas although additional sorbent will reduce the frequency of sorbent exchanges, it will add extra weight [36]. Thus, designers have to take care to balance what weight patients are prepared to carry around versus the inconvenience of sorbent exchange. Taking these considerations into account, the AWAK design has two proposed versions, one weighing around 1 kg and the other 3 kg, depending on the difference in the size of the sorbent cartridges. Replacing the sorbents currently requires the patient to drain out peritoneal dialysate and then re-instil fresh dialysate with each sorbent exchange. Thus, it is important that the sorbents last for at least 24 h to prevent the patient having to perform additional PD exchanges.

Clinical trials aimed at testing the capacity of the current sorbents are expected in 2015. Not surprisingly, the recent enthusiasm for developing wearable and portable dialysis devices has sparked new interest and research into a new generation of more effective and lighter weight sorbents [37].

### Wearable ultrafiltration and haemofiltration devices

**Fig. 3:**
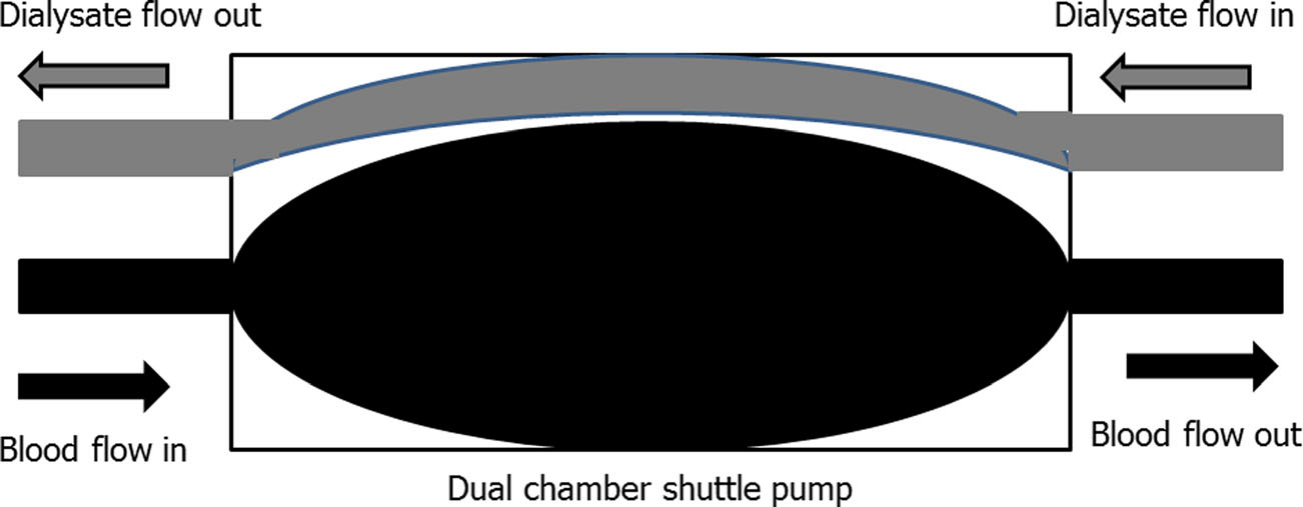
A prototype wearable haemofiltration device using a plasma haemofilter silica-based nanoclay sorbent

The advent of dialysers with increased hydraulic permeability [2] led to the development of wearable and portable haemofiltration designs. However, for haemofiltration to provide effective clearance, large ultrafiltration volumes with the corresponding return of large volumes of a replacement fluid are required. These technical difficulties led to the abandonment of the first generation of wearable haemofiltration devices [38] or resulted in devices being limited to providing low volume ultrafiltration for the treatment of refractory heart failure rather than for treatment of end-stage kidney disease [39]. More recently, a new design based on passing a plasma ultrafiltrate through a silica-based nanoclay sorbent has been developed (Fig. 3) [40], with the majority of plasma ultrafiltrate being returned (to the patient), but some expelled to control fluid balance. As yet, clinical trials of this prototype have been limited to large animal studies with goats. More work is required to refine this design and to determine the capacity of the silica-based nanoclay sorbents.

### Wearable HD devices

**Fig. 4:**
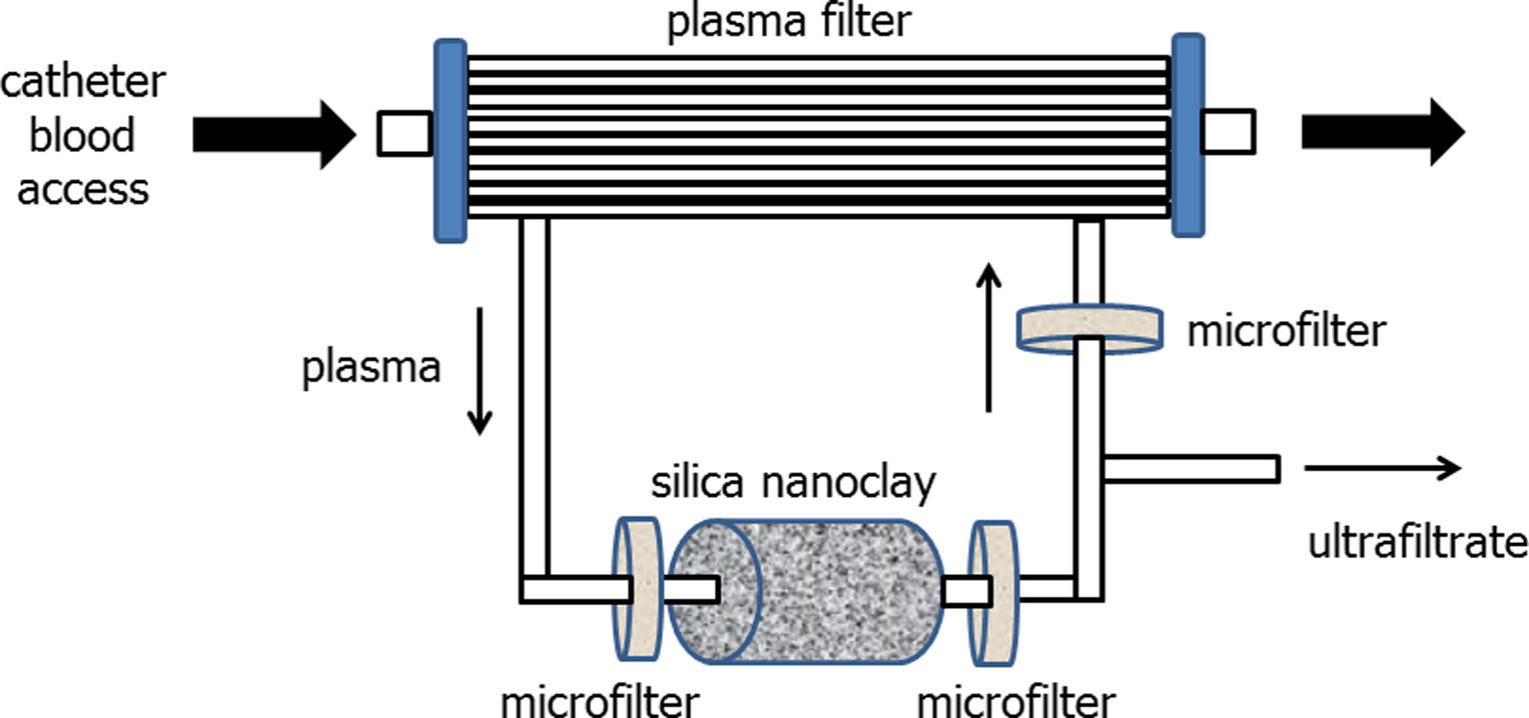
The wearable artificial kidney (WAK) utilises a dual chamber shuttle pump which either pumps blood or dialysate in a counter-current direction

**Fig. 5:**
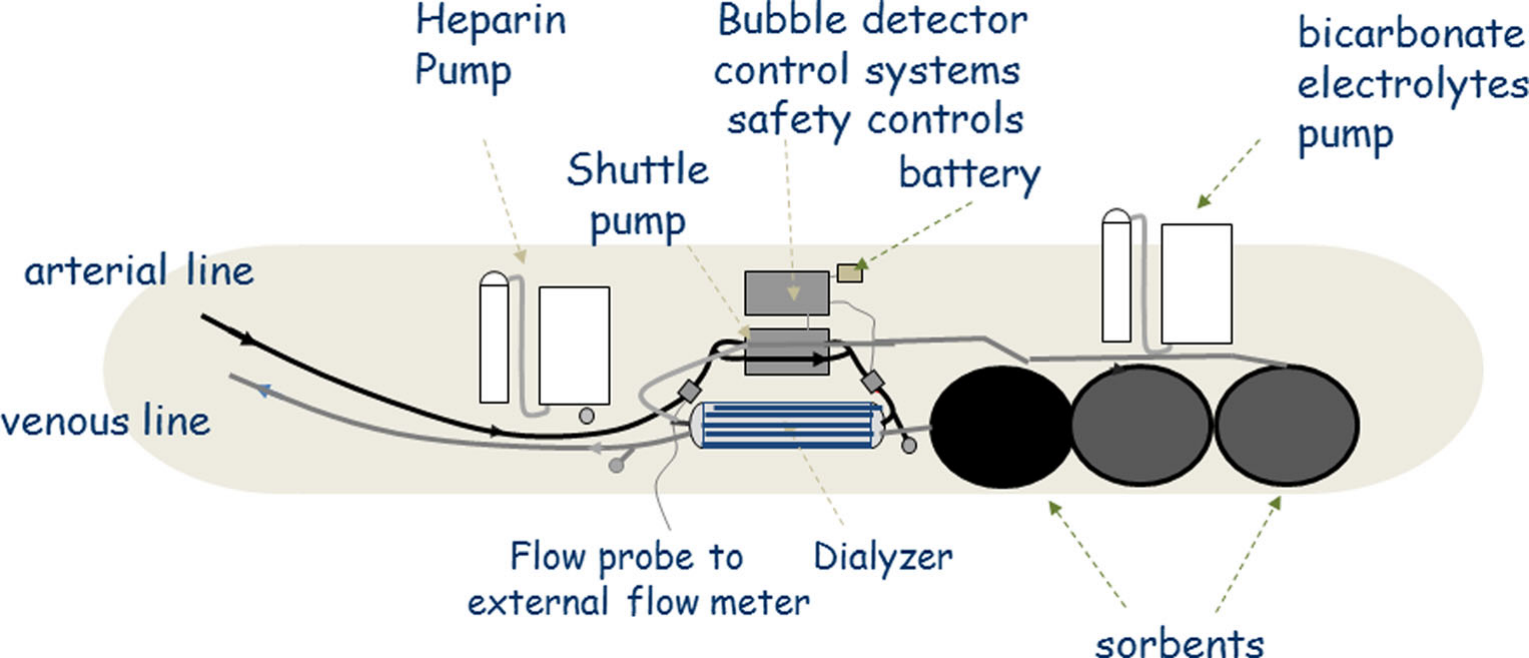
The WAK has both a blood side and a dialysate side, with spent dialysate regenerated by passage through a series of sorbents, followed by the addition of bicarbonate and electrolytes

The advent of nanotechnology manufacturing techniques coupled with miniaturisation and computer technology has allowed the development of experimental wearable HD devices [41]. Current designs are based on the concept of pumping blood and dialysate in a counter-current direction through a standard high-flux dialyser [42]. A single dual chamber battery powered shuttle pump was designed in order to reduce weight and power requirements (Fig. 4). The standard HD machine blood pump produces an almost constant blood flow into the dialyser at a relatively constant pressure. In contrast, the dual chamber shuttle pump pumps blood and dialysate at almost equal flow rates of around 50 ml/min, but as this pump either pumps blood or dialysate, it produces oscillating pressure gradients across the dialyser membrane [43]. The standard HD session is based on delivering a highly efficient but short duration treatment, so although protein deposition on the dialyser membrane and membrane fouling reduce treatment efficiency somewhat, this is not clinically relevant in routine practice. However, for the lower efficiency wearable device which produces much lower creatinine and urea clearances, i.e. of approximately 20–30 ml/min [44], then membrane fouling could potentially markedly reduce efficiency over time. As such, one of the key designs behind the WAK is the shuttle pump, which by generating a pulsatile flow across the dialyser membrane, minimises dialyser membrane protein deposition, thereby maintaining solute clearances over time [45]. A low-sodium sterile dialysate is pumped through the dialyser and then through a series of sorbents in the order of activated microporous carbon, followed by urease to remove urea and then by a number of zirconium-containing sorbents to remove ammonium and hydrogen ions. As these latter sorbents are in effect ion exchangers, they will then release bicarbonate and sodium. Before returning through the dialyser, the dialysate needs to be refreshed by adding bicarbonate, sodium, calcium and magnesium (Fig. 5) [44]. Currently the WAK has only been worn by patients for up to 8 h. Consequently, new trials of treating patients for 24 h are planned later this year to determine the capacity of the sorbents and the composition and requirements of the electrolyte refreshing solution, as these are likely to vary between patients.

As with the AWAK, microbubbles of carbon dioxide develop within the extracorporeal circuit, both in the dialysate and blood compartments. Whereas the conventional HD circuit has an arterial expansion chamber and venous bubble chamber which can accommodate microbubbles, the WAK has no such chambers and, therefore, parts of the plastic tubing in the circuit have been designed using water-impermeable but gas-permeable plastics [43].

The main disadvantage of a wearable HD device is that there is a risk of clotting in the extracorporeal circuit. In the intensive care setting many continuous forms of renal replacement therapy have to be replaced due to circuit clotting [46]. Although there are differences in the balance of pro- and anticoagulants between critically ill patients with acute kidney injury and those with end stage kidney disease [47, 48], clotting in the extracorporeal circuit remains a major hurdle to overcome. It is important to design the blood circuit to minimise areas of turbulence and stagnation, as well as blood–air interfaces as these promote clotting. Equally important is dialyser design and priming to minimise air and microbubble entrapment during priming and operating. Appropriate selection of the central venous access catheter and dialyser design [49], coupled with the dual chamber pump, can all help towards reducing the risk of clotting in the extracorporeal circuit. Currently, a bolus followed by a continuous infusion of unfractionated heparin has been used to anticoagulate patients using the WAK. Repeated exposure to unfractionated heparin has been reported to lead to osteoporosis; in addition, patients differ in heparin requirements. Other alternatives, including repeated daily bolus injections of low-molecular-weight heparins or of heparinoids which have a much longer half-life, have not been explored. The ideal extracorporeal anticoagulant would be an oral medication that had predictable effects and so would not require regular monitoring and, preferably, not be a systemic anticoagulant. Currently only oral systemic anticoagulants are available. Consequently, such future designs of wearable HD devices may require alternative anticoagulant strategies [50, 51], such as oral anti-thrombin or anti-factor Xa inhibitors.

### Implantable HD devices

The commercial market for wearable and portable dialysis devices would be limited to the more active and self-reliant patient, whereas an implantable device could potentially be made available to all dialysis patients. However, any implantable device not only has to have minimal risks for insertion but also has to operate effectively and not fail prematurely. Currently there are no implantable devices undergoing trials, but research is underway to overcome the three main hurdles faced by an implantable dialysis device. Implanting a device between the iliac arteries and veins has the advantage of not requiring a blood pump. Although arterial grafts have been a major success in treating patients with arterial vascular disease, arterio-venous grafts have not been as successful for HD access due to an increased risk of graft thrombosis. As such, thrombus of access darts has been a major hurdle to overcome [52], although polyethylene glycol coating of silicone vascular access darts can reduce or even prevent thrombus formation in the short term [52, 53]. These access darts provide a high blood flow which would lead to an increased risk of protein deposition and clotting if used with conventional haemodialyser designs. Different dialyser designs based on the glomerular basement membrane, which produces a large volume ultrafiltrate from a high-pressure arteriolar input, have been developed using ultrathin silicone slit membranes, similar to a storm drain in the street [54]. In the healthy kidney, although a large volume of ultrafiltrate is produced by the glomerulus, there is then a highly specialised renal tubule designed to selectively reabsorb most of the glomerular filtrate. So although design technology could come up with an equivalent artificial glomerulus design, the search is still on for an equivalent of the human renal tubule. The reverse osmosis water purification system uses a tightly coiled membrane to separate domestic tap water into waste water and a much smaller amount of water for dialysis. This principle could be used to treat the large volume of filtrate, i.e. to reduce the volume, but it would not provide the highly selective capacity provided by the renal tubule in terms of which solutes to conserve and which to discard. A more futuristic approach would be to try and develop an artificial cell-based renal tubule, but even so the function and cell types in the human renal tubule differ from segment to segment of the tubule [55].

## Summary

The concept of wearable and portable dialysis devices dates back to the pioneering days of the 1970s. However, it is only recently with the more recent advances in nanotechnology manufacturing processes, miniaturisation and computer technology has it been possible to develop a number of wearable and portable devices based on PD, haemofiltration and HD. The current generation of wearable devices weigh between 1–3 kg, as there is a balance between sorbent life and sorbent exchanges. As such the weight of the devices may well restrict their use. However, this renewed interest in wearable devices has equally led to improvements in sorbent technology that hopefully will lead to lighter weight devices and underpins the potential success of these devices. We therefore look forward to the development of a newer generation of dialysis devices which could potentially substantially improve the quality of life of the patient with CKD.
